# Author Correction: Assembly of hundreds of novel bacterial genomes from the chicken caecum

**DOI:** 10.1186/s13059-021-02284-4

**Published:** 2021-02-12

**Authors:** Laura Glendinning, Robert D. Stewart, Mark J. Pallen, Kellie A. Watson, Mick Watson

**Affiliations:** 1 Genetics and Genomics, The Roslin Institute and Royal (Dick) School of Veterinary Studies, University of Edinburgh, Edinburgh, Midlothian UK; 2 Microbes in the Food Chain, Quadram Institute Bioscience, Norwich, UK; 3 School of Biological Sciences, University of East Anglia, Norwich, Norfolk UK; 4 School of Veterinary Medicine, University of Surrey, Guildford, Surrey UK

**Correction to: Genome Biol 21, 34 (2020)**

**https://doi.org/10.1186/s13059-020-1947-1**

Following publication of the original paper [1], the authors have reported several typographical and linguistic errors in the new *Candidatus* names presented in Additional file 5: Dataset S4. In addition, the presentation of the names without full protologues has precluded their registration in the *International Journal of Systematic and Evolutionary Microbiology*, in line with the recommendations of the *International Code of Nomenclature of Prokaryotes*. The original Additional File 5 has therefore been replaced by Additional File 5A, which presents Dataset S4 with corrected names, and by Additional File 5B, which contains protologues for the new *Candidatus* names.

## Supplementary Information

**Additional file 5 **A: Clustering of samples at 60% AAI to form genus clusters. Novel genera were defined as clusters of MAGs at 60% AAI which were not assigned a genus by GTDB-Tk. B: Protologues for the new *Candidatus* names.
