# Supplementary material for: Assembly of hundreds of novel bacterial genomes from the chicken caecum
Source: Genome Biol. 2020 Feb 12;21:34. doi: 10.1186/s13059-020-1947-1 (PMC7014784; doi:10.1186/s13059-020-1947-1)
Supplement: Supplementary file 1 — Additional file 1. Contains Figure S1 and S2 (including legends). Also contains Tables S1 to S5. [file 13059_2020_1947_MOESM1_ESM.pdf]

Fig S1:

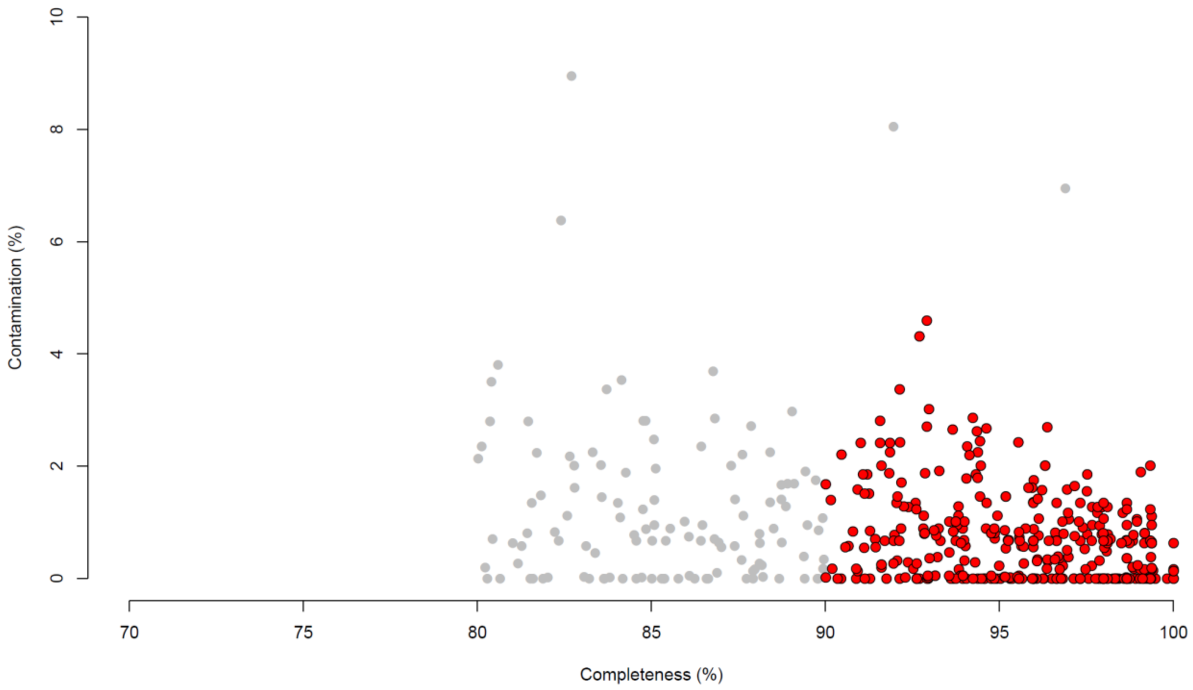

Contamination and completeness of 469 dereplicated draft microbial genomes from the chicken caeca. Contamination and completeness were defined by the CheckM software. Red circles indicate genomes which are >90% complete with <5% contamination. Grey circles indicate genomes which are 80-90% complete with 5-10% contamination.

Fig. S2:

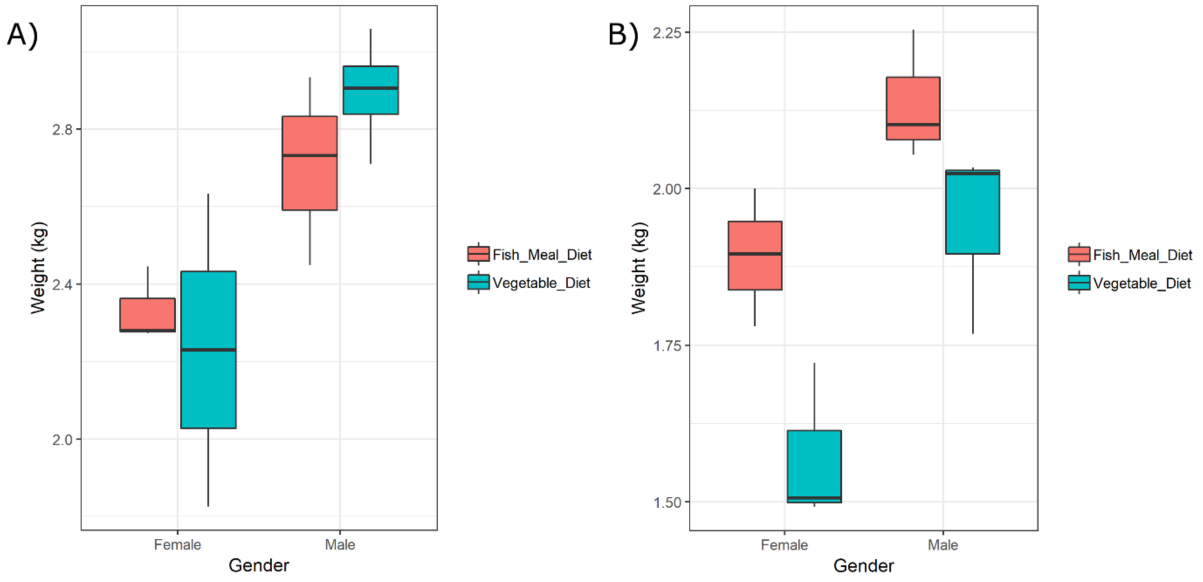

Boxplots showing the weight of birds from either the Ross 308 (n=12) or Ranger Classic (n=12) line, fed either a vegetable diet or a diet which included fish meal.

Table S1: Chicken details

ID	Tag	Line	Diet	Gender	Body weight (kg)
Chicken_1	1472	Ranger Classic	Fish	Female	1.90
Chicken_2	1677	Ross 308	Fish	Male	2.73
Chicken_3	1535	Ranger Classic	Vegetable	Male	2.03
Chicken_4	1591	Ross 308	Vegetable	Male	2.71
Chicken_5	1445	Ranger Classic	Fish	Female	2.00
Chicken_6	1724	Ross 308	Fish	Female	2.28
Chicken_7	1545	Ranger Classic	Vegetable	Male	2.02
Chicken_8	1774	Ross 308	Vegetable	Male	2.93
Chicken_9	1531	Ranger Classic	Fish	Male	2.05
Chicken_10	1738	Ross 308	Fish	Female	2.27
Chicken_11	1498	Ranger Classic	Vegetable	Female	1.49
Chicken_12	1687	Ross 308	Vegetable	Female	1.83
Chicken_13	1423	Ranger Classic	Fish	Female	1.78
Chicken_14	1675	Ross 308	Fish	Male	2.45
Chicken_15	1494	Ranger Classic	Vegetable	Female	1.72
Chicken_16	1707	Ross 308	Vegetable	Male	2.88
Chicken_17	1473	Ranger Classic	Fish	Male	2.10
Chicken_18	1764	Ross 308	Fish	Male	2.93
Chicken_19	1418	Ranger Classic	Vegetable	Male	1.768
Chicken_20	1730	Ross 308	Vegetable	Male	3.058
Chicken_21	1421	Ranger Classic	Fish	Male	2.254
Chicken_22	1640	Ross 308	Fish	Female	2.446
Chicken_23	1507	Ranger Classic	Vegetable	Female	1.506

Chicken_24	1568	Ross 308	Vegetable	Female	2.634

Table S2: Formulation of fish meal and vegetable starter diets (Target Feeds Ltd, UK)

Raw materials	Broiler starter fish (%)	Broiler starter standard (vegetable) (%)
1 Barley raw ground	12.7	10.5
10 Wheat raw ground	58.4	50.0
423 Soya ext hipro	10.5	26
426 Full fat soya masham	5	5
459 Provimi white fishmeal	10	0
712 L-lysine Hcl	0.35	0.4
713 DL methionine	0.33	0.4
714 L-threonine	0.15	0.15
716 L-Tryptophan	0.02	0.00
810 Soya oil	1.6	4
896 Limestone flour tru. 270	0.25	1.25
901 Monocalcium phosphate	0.1	1.5
904 Salt	0.05	0.25
906 Sodium bicarbonate	0.15	0.15
3482 Br. Trials PMX. Starter 4K	0.4	0.4

Table S3: Formulation of fish meal and vegetable grower diets (Target Feeds Ltd, UK)

**Raw materials**	**Broiler grower fish (%)**	**Broiler grower standard (vegetable) (%)**
1 Barley raw ground	8.4	8.4
10 Wheat raw ground	60.34	55.0
423 Soya ext hipro	15.0	23.0
425 Full fat soya Cherwell	5.0	5.0
459 Provimi white fishmeal	5.0	0.0
712 L-lysine Hcl	0.3	0.3
713 DL methionine	0.31	0.35
714 L-threonine	0.15	0.15
810 Soya oil	3.25	4.5
900 Limestone Trucal 52	0.75	1.25
901 Monocalcium phosphate	0.7	1.25
904 Salt	0.25	0.25
906 Sodium bicarbonate	0.15	0.15
3483 Br. Trials PMX. Gro/Fin 4K	0.4	0.4

Table S4: Nutritional info of fish meal and vegetable diets (Target Feeds Ltd, UK)

Nutrient	Broiler starter fish	Broiler starter standard (vegetable)	Broiler grower fish	Broiler grower standard (vegetable)
Oil EE	4.5246	6.3685	5.8789	6.8528
Protein	21.7423	21.4436	20.2641	20.1993
Fibre	2.9826	3.1325	2.9279	3.0280
Ash	4.7791	6.0665	5.2399	5.6815
Me-P	12.7907	12.7703	13.0385	13.0441
Tlysine	1.4372	1.4343	1.2819	1.2682
Avlysine	1.3230	1.3356	1.1894	1.1829
Meth	0.6928	0.6900	0.6209	0.6260
M+C	0.9902	1.0155	0.9164	0.9373
Threo	0.9132	0.9013	0.8504	0.8497
Trypt	0.2509	0.2523	0.2235	0.2359
Calcium	0.9549	0.9805	0.9417	0.9304
Phos	0.6831	0.7289	0.6658	0.6575
Av phos	0.4908	0.4818	0.4598	0.4227
Salt	0.2991	0.3092	0.4024	0.3077
Sodium	0.1677	0.1749	0.2128	0.1763
Vitamin A	13.5000	13.5000	10.0000	10.0000
Vitamin D3	5.0000	5.0000	5.0000	5.0000
Vitamin E	100.0000	100.0000	100.0000	100.0000

Table S5: DNA extractions

ID	Sampling_date	DNA_extraction_date	DNA_extraction_batch
Chicken_4	27th June 2018	28th June 2018	1
Chicken_8	27th June 2018	6th July 2018	2
Chicken_12	27th June 2018	6th July 2018	2
Chicken_16	27th June 2018	28th June 2018	3
Chicken_20	27th June 2018	28th June 2018	3
Chicken_24	27th June 2018	28th June 2018	1
Chicken_2	27th June 2018	6th July 2018	2
Chicken_6	27th June 2018	28th June 2018	3
Chicken_10	27th June 2018	6th July 2018	2
Chicken_14	27th June 2018	6th July 2018	2
Chicken_18	27th June 2018	28th June 2018	3
Chicken_22	27th June 2018	6th July 2018	2
Chicken_3	27th June 2018	6th July 2018	2
Chicken_7	27th June 2018	28th June 2018	1
Chicken_11	27th June 2018	28th June 2018	1
Chicken_15	27th June 2018	28th June 2018	3
Chicken_19	27th June 2018	28th June 2018	3
Chicken_23	27th June 2018	28th June 2018	1
Chicken_1	27th June 2018	28th June 2018	1
Chicken_5	27th June 2018	28th June 2018	3
Chicken_9	27th June 2018	28th June 2018	1
Chicken_13	27th June 2018	6th July 2018	2
Chicken_17	27th June 2018	28th June 2018	1

Chicken_21	27th June 2018	28th June 2018	3
