# Supplementary material for: Assembly of hundreds of novel bacterial genomes from the chicken caecum
Source: Genome Biol. 2020 Feb 12;21:34. doi: 10.1186/s13059-020-1947-1 (PMC7014784; doi:10.1186/s13059-020-1947-1)
Supplement: Supplementary file 8 — Additional file 8. Review history. [file 13059_2020_1947_MOESM8_ESM.docx]

Review History

**First round of review**

**Reviewer 1**

**Comments to author:**

This is technically nice paper which considerably extends knowledge on genomes of chicken caecal microbiota. This manuscript also expands original paper from this group Sergeant et al, Extensive microbial and functional diversity within the chicken caecal microbiome. PLoS One 2014;9:13). Naturally, it is limited by limits of metagenomic sequencing itself, for example authors cannot provide something so trivial like Gram staining of all newly described bacteria or, though here I am not 100 % certain, they probably cannot present 16 rRNA sequences for each of newly identified strain. Furthermore, authors sometimes little bit ignore common biology.

I have only a few specific comments and some general comments for authors consideration though the general comments mostly cannot be used for the manuscript improvement but can be considered when preparing follow up papers.

l. 36, popularity of chicken meat is globally is influenced the most by the fact that all major religions tolerate chickens while Muslims refuse pork or Hindu refuse beef.

l.47, say that "mostly from carbohydrates" since these acids can be formed also from amino acid with butyrate production by lysine fermentation present in Pseudoflavonifractor, Odoribacter or Butyricimonas as just one example

l.65 you may check and refer to a recent paper  Crhanova et al, Systematic Culturomics Shows that Half of Chicken Caecal Microbiota Members can be Grown in Vitro Except for Two Lineages of Clostridiales and a Single Lineage of Bacteroidetes. Microorganisms 2019, 7(11), 496. It seems that the culture is not that difficult as originally thought.

l. 74, 75, 79, 81, use past tense

l. 76, why have you decided for broilers. Remember that chickens reach sexual maturity around week 18 of life and may live for 15-20 years. And you sample 30 days old chickens and want to determine chicken core microbiota. By this decision you missed much more interesting bacteria like all Bacteroidetes species, Acidamimococcacea, Veillonellaceae, and genera from Proteobacteria such as Desulfovibrio, Succinatomas, Anaerobiospirillum. These are taxons which belong to chicken core microbiota many of those which you recorded (e.g. Gordonibacter), these will disappear when full chicken adapted microbiota would appear. My strong recommendation is to use at least 30-week-old egg layers and test the purified DNA bys 16S sequencing that Bacteroides to Firmicutes ratio is around 50 to 50, and select such samples instead on naive and immature broilers.

l. 89, Dataset 1, is fine but... I in my analyses you statistics and complicated approaches to a considerably lesser extent than you. But I use more common sense, biological way of thinking. One of my controls is to use dot plots in Excel. Use dataset1 and prepare a dot plot of CMAG_9 against CMAG_10. The correlation cannot be more linear and my conclusion is that these to CMAGs belong to the same bacterium. These are not two different strains. These two CMAGs belong to Pseudoflavonifractor and I cannot biologically imagine such positive correlation in closely related species. Closely related species with similar life style would be competitors, I can imagine negative correlation but not positive. This points towards limits of metagenomics. And this is not the only case. The number of different CMAGs is therefore lower than you report and being aware of such potential mistakes in your algorithms, you have to reduce strength of all of you conclusions. You have to have pure cultures.

l.94, define ANI abbreviation

l. 102, dataset 2, the fact that except for a single Alistipes you did not sequence any other Bacteroidetes species indicates mistaken animal selection. You forgot for basal and simple biology and worked in a "sterile", academic fashion. I do not mean this in an arrogant or aggressive way but simply it is a pity...

l. 142, AAI, define the abbreviation
l. 143, if possible, do not start sentences with numbers
l.144, you should remind that your system classify family Ruminococcaceae into Oscillispirales
l. 146, new species, well, considering potential mistakes in metagenomic sequences, so trivial that I could find them after 5 minute visual check of your dataset 1, I do not recommend this. We are preparing manuscripts on new bacterial species and one of requirements is that the strain has to be deposited in 3 different publicly available culture collections. You even do not show 16S rRNA sequence. So this is really quite self-confident. Please, reconsider.
l. 172, well, since you sequenced 30 days old chickens, I do not think that you can conclude on core chicken microbiota. Where is Bacteroides ceacigallinarum? Megamonas hypermegale? Succinatomonas hippei? Desulfovibrio piger? I do not know. This only a core microbiota of chickens kept in your animal facilities and the fact that some of them you detected also in other samples does not mean that much since you did not test in these other samples common chicken microbiota members which were absent in your broilers.

All the discussion on enrichment of particular MAGs in different chicken lines and feed. We have performed hundreds of experiments with oral inoculation of chicks with different pre or probiotics, and characterised caecal microbiota. Quite recently we computed for our own purposes microbiota composition only of control chicks, 17 different control groups with each group consisting of 4 or 5 chicks. PCoA plot resulted in 17 clusters depending on experiment. When I realise that there might be errors in the metagenomic data evaluation and since you did not repeat the experiment, please be much more reserved and critical. Especially very young chicks can be colonised by many bacteria from environment (see also very recent paper Kubasova et al, Gut Anaerobes Capable of Chicken Caecum Colonisation. Microorganisms 2019, 7, 597), and it is probable that you report here many biologically irrelevant observations.

l. 302, as already said, this is quite biased. If you too much better suited sample, i.e. adult hens in the middle of egg lay, you would end up in pretty different core microbiota - but more relevant for Gallus gallus

l. 322, there, not those

l. 352 and 353, and also 361, yes, full agreement and therefore also a reason for my reservation to extensive description of differences in microbiota composition of different liens or after different feed regime.

**Reviewer 2**

**Comments to author:**

The manuscript by Glendinning and colleagues describes the metagenomic sequencing of chicken caecal microbiota samples and analysis to derive assembled genomes of hundreds of different bacteria. As well as characterising the genomes of novel strain of bacteria that are well known to be components of the chicken gut microbiota, they have also identified other bacteria which have not been previously recognised. The work is very interesting and makes a great contribution to microbiota studies and microbiology in general. The manuscript is well written, concise, and clear.

It would be useful to include a little more information about the binning of the sequence reads. What percentage of the reads don't end up in a bin or are in bins that don't assemble? What percentage of reads were chicken, parasite, viral, i.e. not bacterial?

Why are a further 92 genomes recovered from the co-assembly? Obviously, the data is in the single samples. Is coverage too low in all the single samples, or what?

The genome assemblies by themselves are more than sufficient to justify publication of this work but the authors have also included a small amount of functional analysis by identifying, enumerating and comparing the CAZymes content of the genomes. This is of some interest but it would be preferable to include some functional analysis to try to explain why some of the newly identified genera have not been cultured - this would be of more immediate benefit to the scientific community.

Minor issues
Ensure there is a space between genus abbreviation and species name (e.g Lns 221, 222, 313, 314, 327, 329, 333.
Ln381. Correct 'receiving' to 'received'

**Authors Response**

**Point-by-point responses to the reviewers’ comments:**

Reviewer #1:

“This is technically nice paper which considerably extends knowledge on genomes of chicken caecal microbiota. This manuscript also expands original paper from this group Sergeant et al, Extensive microbial and functional diversity within the chicken caecal microbiome. PLoS One 2014;9:13).“

*We thank the reviewer for their supportive comments.*

“Naturally, it is limited by limits of metagenomic sequencing itself, for example authors cannot provide something so trivial like Gram staining of all newly described bacteria or, though here I am not 100 % certain, they probably cannot present 16 rRNA sequences for each of newly identified strain. Furthermore, authors sometimes little bit ignore common biology.”

*Due to the nature of metagenomic binning using short reads, we are indeed unable to construct 16 rRNA sequences.*

“I have only a few specific comments and some general comments for authors consideration though the general comments mostly cannot be used for the manuscript improvement but can be considered when preparing follow up papers.”

1. “l. 36, popularity of chicken meat is globally is influenced the most by the fact that all major religions tolerate chickens while Muslims refuse pork or Hindu refuse beef.”

*This has now been added to lines 39-41.*

2. “l.47, say that "mostly from carbohydrates" since these acids can be formed also from amino acid with butyrate production by lysine fermentation present in Pseudoflavonifractor, Odoribacter or Butyricimonas as just one example.”

*This has now been added to line 48.*

3. “l.65 you may check and refer to a recent paper  Crhanova et al, Systematic Culturomics Shows that Half of Chicken Caecal Microbiota Members can be Grown in Vitro Except for Two Lineages of Clostridiales and a Single Lineage of Bacteroidetes. Microorganisms 2019, 7(11), 496. It seems that the culture is not that difficult as originally thought.”

*This reference has been added to line 65.*

4. “l. 74, 75, 79, 81, use past tense”

*Changed (lines 76, 77, 81, 83, 84)*

5. “l. 76, why have you decided for broilers. Remember that chickens reach sexual maturity around week 18 of life and may live for 15-20 years. And you sample 30 days old chickens and want to determine chicken core microbiota. By this decision you missed much more interesting bacteria like all Bacteroidetes species, Acidamimococcacea, Veillonellaceae, and genera from Proteobacteria such as Desulfovibrio, Succinatomas, Anaerobiospirillum. These are taxons which belong to chicken core microbiota many of those which you recorded (e.g. Gordonibacter), these will disappear when full chicken adapted microbiota would appear. My strong recommendation is to use at least 30-week-old egg layers and test the purified DNA bys 16S sequencing that Bacteroides to Firmicutes ratio is around 50 to 50, and select such samples instead on naive and immature broilers.”

*Our research group is currently interested in studying the microbiota of commercial broiler (meat-producing) breeds rather than egg-laying breeds. We agree that understanding the microbiota of egg-laying birds is also of importance but this is already being well researched by other groups, such as that of Dr Ivan Rychlik. We hope that by increasing our understanding of the broiler microbiota we may in the future be able to optimise the microbiota in these birds, leading to improvements in poultry meat production and sustainability.*

*For this study, we selected two Aviagen broiler lines which are commonly used in commercial farms. We agree that if we had investigated chickens of an older age we may have observed different microorganisms. However, while some breeds of chickens can reach 15-20 years of age this is not the case for commercial broiler lines. The Ross 308 line has been bred to have a life span of around 5-6 weeks as this is the time it is usually slaughtered, and even the Ranger Classic, a slower growing line, has been bred to have a lifespan far shorter than the age of chicken sexual maturity. Without feed restriction (which is not carried out on commercial farms) these birds would not survive long past our chosen sampling timepoint. We feel that the microbiota of broilers after this normal slaughter age is relatively unimportant if they rarely live above this age in a commercial setting.*

*While the taxa described by the reviewer are present in abundance in commercial layers, it has previously been demonstrated that they are far less abundant in commercial broilers and in fact the ratio of Bacteroidetes to Firmicutes is similar to what we observe in our study:* [*https://journals.plos.org/plosone/article?id=10.1371/journal.pone.0110076*](https://journals.plos.org/plosone/article?id=10.1371/journal.pone.0110076)*. This has also been observed in various other studies on commercial broiler lines, as we have described in lines 302-304.*

6. “l. 89, Dataset 1, is fine but... I in my analyses you statistics and complicated approaches to a considerably lesser extent than you. But I use more common sense, biological way of thinking. One of my controls is to use dot plots in Excel. Use dataset1 and prepare a dot plot of CMAG_9 against CMAG_10. The correlation cannot be more linear and my conclusion is that these to CMAGs belong to the same bacterium. These are not two different strains. These two CMAGs belong to Pseudoflavonifractor and I cannot biologically imagine such positive correlation in closely related species. Closely related species with similar life style would be competitors, I can imagine negative correlation but not positive. This points towards limits of metagenomics. And this is not the only case. The number of different CMAGs is therefore lower than you report and being aware of such potential mistakes in your algorithms, you have to reduce strength of all of you conclusions. You have to have pure cultures.”

*We agree that metagenomics has limitations and that pure cultures remain the gold standard. We have changed the wording in our conclusion to reflect this (lines 382-384). However, we follow best practice workflows in our production of MAGs, with rigorous quality control standards as described in our methods and as followed by several other publications (Passoli et al. Cell 2019, Almeida et al. Nature 2019, Stewart et al. Nature Communications 2018, Stewart et al. Nature Biotechnology 2019).*

*We do not feel confident that using such correlation methods as described by the reviewer would improve our MAG production as we believe it may lead to us removing genuine results from our dataset. Contrary to the reviewer’s claims, there are previous examples of similar strains/species within microbial communities being present in the same microbiota communities (*[*https://www.sciencedirect.com/science/article/pii/S0092867416303993*](https://www.sciencedirect.com/science/article/pii/S0092867416303993)*,* [*https://microbiomejournal.biomedcentral.com/articles/10.1186/s40168-019-0681-y*](https://microbiomejournal.biomedcentral.com/articles/10.1186/s40168-019-0681-y)*). Indeed, microbes are more likely to colonise as part of a microbial community which already contains representatives which are taxonomically closely related to them (*[*https://journals.plos.org/plospathogens/article?id=10.1371/journal.ppat.1000711*](https://journals.plos.org/plospathogens/article?id=10.1371/journal.ppat.1000711)*) and closely related transplanted strains do not vary in their level of engraftment (*[*https://www.cell.com/cell-host-microbe/pdf/S1931-3128(18)30038-6.pdf*](https://www.cell.com/cell-host-microbe/pdf/S1931-3128(18)30038-6.pdf)*).*

7. “l.94, define ANI abbreviation”

*Definition added to line 129.*

8. “l. 102, dataset 2, the fact that except for a single Alistipes you did not sequence any other Bacteroidetes species indicates mistaken animal selection. You forgot for basal and simple biology and worked in a "sterile", academic fashion. I do not mean this in an arrogant or aggressive way but simply it is a pity...”

*We disagree that this lack of Bacteroidetes indicates a mistaken selection of animals. Please see our response to comment number 5.*

9. “l. 142, AAI, define the abbreviation”

*Definition added to line 148.*

10. “l. 143, if possible, do not start sentences with numbers

*Changed (line 149).*

11. “ l.144, you should remind that your system classify family Ruminococcaceae into Oscillispirales”

*Added (line 150).*

12. “l. 146, new species, well, considering potential mistakes in metagenomic sequences, so trivial that I could find them after 5 minute visual check of your dataset 1, I do not recommend this. We are preparing manuscripts on new bacterial species and one of requirements is that the strain has to be deposited in 3 different publicly available culture collections. You even do not show 16S rRNA sequence. So this is really quite self-confident. Please, reconsider.”

*For the reviewer’s comments on errors, please see our response to comment number 6.*

*We disagree with the definition of a strain/species as described by the reviewer as we feel it ignores the valuable research being done using culture-free techniques. Using this traditional method of species identification, if a microbe were truly unculturable then it would never be able to be assigned a species name, even if we possessed a highly-complete, high-quality genome. It has been demonstrated that using average nucleotide identity (ANI) is a robust means of identifying species boundaries (*[*https://www.nature.com/articles/s41467-018-07641-9*](https://www.nature.com/articles/s41467-018-07641-9)*) and it is now common practice to describe uncultured taxa as novel based on their nucleotide similarity to other publically available genomes (*[*https://www.nature.com/articles/ismej201178*](https://www.nature.com/articles/ismej201178)*,* [*https://bmcgenomics.biomedcentral.com/articles/10.1186/s12864-017-4294-1*](https://bmcgenomics.biomedcentral.com/articles/10.1186/s12864-017-4294-1)*,* [*https://bmcgenomics.biomedcentral.com/articles/10.1186/s12864-016-3055-x*](https://bmcgenomics.biomedcentral.com/articles/10.1186/s12864-016-3055-x)*,* [*https://genomebiology.biomedcentral.com/articles/10.1186/s13059-015-0834-7*](https://genomebiology.biomedcentral.com/articles/10.1186/s13059-015-0834-7)*,* [*https://www.sciencedirect.com/science/article/pii/S0092867419300017*](https://www.sciencedirect.com/science/article/pii/S0092867419300017)*,* [*https://www.nature.com/articles/s41586-019-0965-1*](https://www.nature.com/articles/s41586-019-0965-1)*).*

13. “l. 172, well, since you sequenced 30 days old chickens, I do not think that you can conclude on core chicken microbiota. Where is Bacteroides ceacigallinarum? Megamonas hypermegale? Succinatomonas hippei? Desulfovibrio piger? I do not know. This only a core microbiota of chickens kept in your animal facilities and the fact that some of them you detected also in other samples does not mean that much since you did not test in these other samples common chicken microbiota members which were absent in your broilers.”

*This section describes the core microbiota we found within our dataset (as specified in lines 185-186). We do not aim to describe the entire core microbiota of all chickens; if this were our aim then we would agree with the reviewer that we should also look for the bacteria they have named. Instead, our aim was to identify whether the bacteria we have identified are part of a core microbiota. We therefore demonstrated that our samples are core members of the chicken microbiota in European broiler farms by showing that they occur in the majority of samples from previously sequenced data. This demonstrates that our microbes do not only represent part of a core microbiota from only our facility but are present on commercial farms in multiple countries. We agree with the reviewer that we can’t claim that they represent a core chicken microbiota as we have only examined broilers. We have therefore changed “chicken” to “broiler” in lines 171, 179 and 315.*

14. “All the discussion on enrichment of particular MAGs in different chicken lines and feed. We have performed hundreds of experiments with oral inoculation of chicks with different pre or probiotics, and characterised caecal microbiota. Quite recently we computed for our own purposes microbiota composition only of control chicks, 17 different control groups with each group consisting of 4 or 5 chicks. PCoA plot resulted in 17 clusters depending on experiment. When I realise that there might be errors in the metagenomic data evaluation and since you did not repeat the experiment, please be much more reserved and critical. Especially very young chicks can be colonised by many bacteria from environment (see also very recent paper Kubasova et al, Gut Anaerobes Capable of Chicken Caecum Colonisation. Microorganisms 2019, 7, 597), and it is probable that you report here many biologically irrelevant observations.”

*We have urged caution about generalising our results in lines 309-314.  We have also urged further caution in lines 322-324.*

15. “l. 302, as already said, this is quite biased. If you too much better suited sample, i.e. adult hens in the middle of egg lay, you would end up in pretty different core microbiota - but more relevant for Gallus gallus”

*This has been changed to reflect that we are only discussing the core microbiota within our dataset (line 309).*

16. “l. 322, there, not those”

*Changed (line 331).*

17. “l. 352 and 353, and also 361, yes, full agreement and therefore also a reason for my reservation to extensive description of differences in microbiota composition of different liens or after different feed regime.”

We thank the author for their support of our comments. We feel that even with these reservations it is worth acknowledging the significant differences between our groups, as long as these reservations are made clear.

Reviewer #2:

“The manuscript by Glendinning and colleagues describes the metagenomic sequencing of chicken caecal microbiota samples and analysis to derive assembled genomes of hundreds of different bacteria. As well as characterising the genomes of novel strain of bacteria that are well known to be components of the chicken gut microbiota, they have also identified other bacteria which have not been previously recognised. The work is very interesting and makes a great contribution to microbiota studies and microbiology in general. The manuscript is well written, concise, and clear.”

*We would like to thank the reviewer for their supportive comments.*

1. “It would be useful to include a little more information about the binning of the sequence reads. What percentage of the reads don't end up in a bin or are in bins that don't assemble? What percentage of reads were chicken, parasite, viral, i.e. not bacterial?”

*We have added more information on the percentage of reads which were assembled and binned (lines 409-410, 414-415 and 420-421). We have also added a Kraken analysis to calculate the proportion of reads which were not bacterial in origin (methods: lines 406-407, results: lines 94-96).*

2. “Why are a further 92 genomes recovered from the co-assembly? Obviously, the data is in the single samples. Is coverage too low in all the single samples, or what?”

*Co-assemblies allow us to construct genomes which exist in lower coverage than those which could be assembled via single sample binning (explanation added to lines 97-99).*

3. “The genome assemblies by themselves are more than sufficient to justify publication of this work but the authors have also included a small amount of functional analysis by identifying, enumerating and comparing the CAZymes content of the genomes. This is of some interest but it would be preferable to include some functional analysis to try to explain why some of the newly identified genera have not been cultured - this would be of more immediate benefit to the scientific community.”

*We do not believe that the microbes we have identified are necessarily unculturable. In fact, based upon recent publications (as described by reviewer 1) we think it likely that many of them will be culturable, it is merely that the correct culturing conditions have not previously been used. We hope that the CAZyme information we provide about these organisms will help provide more information about their preferred substrates and will therefore aid in future culturing efforts.*

4. “Ensure there is a space between genus abbreviation and species name (e.g Lns 221, 222, 313, 314, 327, 329, 333.”

*Added (lines 227-228, 229, 320-321, 336, 338 and 343).*

5. “Ln381. Correct 'receiving' to 'received'”

*Corrected (line 393).*

**Second round of review**

**Reviewer 1**

Authors accepted and included majority of my suggestions into amended version of the manuscript and where we disagree, well, this happens. Good paper.

**Reviewer 2**

Thanks for addressing the reviewers comments. This is a nice paper that will be of considerable interest to all working in chicken microbiota and animal microbiota in general.
