# Supplementary material for: Author Correction: Assembly of hundreds of novel bacterial genomes from the chicken caecum
Source: Genome Biol. 2021 Feb 12;22:60. doi: 10.1186/s13059-021-02284-4 (PMC7879605; doi:10.1186/s13059-021-02284-4)
Supplement: Supplementary file 1 — Additional file 5 A: Clustering of samples at 60% AAI to form genus clusters. Novel genera were defined as clusters of MAGs at 60% AAI which were not assigned a genus by GTDB-Tk. B: Protologues for the new Candidatus names. [file 13059_2021_2284_MOESM1_ESM.zip › 13059_2020_1947_MOESM5B_ESM_5B.docx]

**Description of *Candidatus* Adamsella gen. nov.**

*Candidatus* Adamsella (A.dams.el'la. N.L. fem. n. *Adamsella* named in honour of David Adams, British microbiologist recognized for his contribution to the study of cyanobacteria).

A bacterial genus identified by metagenomic analyses of the chicken caecum. The genus includes all bacteria with genomes that show ≥60% average amino acid identity (AAI) to the genome of the type strain from the type species *Candidatus* Adamsella avium. GTDB-Tk/GTDB Release 86 assigns this genus to the order *Gastranaerophilales*.

**Description of *Candidatus* Adamsella avium sp. nov.**

*Candidatus* Adamsella avium (a'vi.um. L. gen. pl. n. *avium* of birds).

A bacterial species identified by metagenomic analyses of the chicken caecum. This species includes all bacteria with genomes that show ≥95% average nucleotide identity (ANI) to the type genome for the species, which is available via NCBI Assembly GCA_904420445.1 and to which we have assigned the MAG ID Chicken_20_mag_183. The GC content of the type genome is 37.4% and the genome length is 2.10 Mbp.

**Description of *Candidatus* Alangreenwoodia gen. nov.**

*Candidatus* Alangreenwoodia (A.lan.green.wood'i.a. N.L. fem. n. *Alangreenwoodia* named after Alan Greenwood for his contribution to avian biology).

A bacterial genus identified by metagenomic analyses of the chicken caecum. The genus includes all bacteria with genomes that show ≥60% average amino acid identity (AAI) to the genome of the type strain from the type species *Candidatus* Alangreenwoodia gallinarii. GTDB-Tk/GTDB Release 86 assigns this genus to the order *Peptostreptococcales* and to the family *Anaerovoracaceae*.

**Description of *Candidatus* Alangreenwoodia gallinarii sp. nov.**

*Candidatus* Alangreenwoodia gallinarii (gal.li.na'ri.i. N.L. neut. n. *gallinarii* of the hen house).

A bacterial species identified by metagenomic analyses of the chicken caecum. This species includes all bacteria with genomes that show ≥95% average nucleotide identity (ANI) to the type genome for the species, which is available via NCBI Assembly GCA_904398315.1 and to which we have assigned the MAG ID Coassembly_mag_499. The GC content of the type genome is 51.4% and the genome length is 2.25 Mbp.

**Description of *Candidatus* Allobutyricicoccus gen. nov.**

*Candidatus* Allobutyricicoccus (Al.lo.bu.ty.ri.ci.coc'cus. Gr. masc. adj. *allos* different; N.L. masc. n. *Butyricicoccus* a bacterial generic name; N.L. masc. n. *Allobutyricicoccus* organism different from but related to the genus *Butyricicoccus*).

A bacterial genus identified by metagenomic analyses of the chicken caecum. The genus includes all bacteria with genomes that show ≥60% average amino acid identity (AAI) to the genome of the type strain from the type species *Candidatus* Allobutyricicoccus pentlandensis. GTDB-Tk/GTDB Release 86 assigns this genus to the order *Oscillospirales* and to the family *Butyricicoccaceae*.

**Description of *Candidatus* Allobutyricicoccus pentlandensis sp. nov.**

*Candidatus* Allobutyricicoccus pentlandensis (pent.land.en'sis. N.L. masc. n. *pentlandensis* named after Pentland Regional Park near where the taxon was first described).

A bacterial species identified by metagenomic analyses of the chicken caecum. This species includes all bacteria with genomes that show ≥95% average nucleotide identity (ANI) to the type genome for the species, which is available via NCBI Assembly GCA_904398645.1 and to which we have assigned the MAG ID Coassembly_mag_96. The GC content of the type genome is 49.7% and the genome length is 1.86 Mbp.

**Description of *Candidatus* Allochristensenella gen. nov.**

*Candidatus* Allochristensenella (Al.lo.chris.ten.sen.el'la. Gr. masc. adj. *allos* different; N.L. fem. n. *Christensenella* a bacterial generic name; N.L. fem. n. *Allochristensenella* organism different from but related to the genus *Christensenella*).

A bacterial genus identified by metagenomic analyses of the chicken caecum. The genus includes all bacteria with genomes that show ≥60% average amino acid identity (AAI) to the genome of the type strain from the type species *Candidatus* Allochristensenella caecavium. GTDB-Tk/GTDB Release 86 assigns this genus to the order *Christensenellales*.

**Description of *Candidatus* Allochristensenella caecavium sp. nov.**

*Candidatus* Allochristensenella caecavium (caec.a'vi.um. N.L. neut. n. *caecum* caecum; L. fem. n. *avis* a bird; N.L. gen. pl. n. *caecavium* from the caeca of birds).

A bacterial species identified by metagenomic analyses of the chicken caecum. This species includes all bacteria with genomes that show ≥95% average nucleotide identity (ANI) to the type genome for the species, which is available via NCBI Assembly GCA_904397865.1 and to which we have assigned the MAG ID Coassembly_mag_524. The GC content of the type genome is 52.4% and the genome length is 1.61 Mbp.

**Description of *Candidatus* Alloclostridium gen. nov.**

*Candidatus* Alloclostridium (Al.lo.clos.tri'di.um. Gr. masc. adj. *allos* different; N.L. neut. n. *Clostridium* a bacterial generic name; N.L. neut. n. *Alloclostridium* organism different from but related to the genus *Clostridium*).

A bacterial genus identified by metagenomic analyses of the chicken caecum. The genus includes all bacteria with genomes that show ≥60% average amino acid identity (AAI) to the genome of the type strain from the type species *Candidatus* Alloclostridium intestinigallinarum. GTDB-Tk/GTDB Release 86 assigns this genus to the order 4C28d-15 and to the family UBA3700.

**Description of *Candidatus* Alloclostridium intestinigallinarum sp. nov.**

*Candidatus* Alloclostridium intestinigallinarum (in.tes.ti.ni.gal.li.na'rum. L. neut. n. *intestinum* gut intestine; L. fem. n. *gallina* a hen; N.L. gen. pl. n. *intestinigallinarum* from the intestine of chickens).

A bacterial species identified by metagenomic analyses of the chicken caecum. This species includes all bacteria with genomes that show ≥95% average nucleotide identity (ANI) to the type genome for the species, which is available via NCBI Assembly GCA_904393845.1 and to which we have assigned the MAG ID Chicken_8_mag_61. The GC content of the type genome is 50.9% and the genome length is 1.86 Mbp.

**Description of *Candidatus* Alloruminococcus gen. nov.**

*Candidatus* Alloruminococcus (Al.lo.ru.mi.no.coc'cus. Gr. masc. adj. *allos* different; N.L. masc. n. *Ruminococcus* a bacterial generic name; N.L. masc. n. *Alloruminococcus* organism different from but related to the genus *Ruminococcus*).

A bacterial genus identified by metagenomic analyses of the chicken caecum. The genus includes all bacteria with genomes that show ≥60% average amino acid identity (AAI) to the genome of the type strain from the type species *Candidatus* Alloruminococcus vanvlietii. GTDB-Tk/GTDB Release 86 assigns this genus to the order *Oscillospirales* and to the family *Ruminococcaceae*.

**Description of *Candidatus* Alloruminococcus vanvlietii sp. nov.**

*Candidatus* Alloruminococcus vanvlietii (van.vliet'i.i. N.L. gen. n. *vanvlietii* named in honour of Dutch microbiologist Arnoud van Vliet).

A bacterial species identified by metagenomic analyses of the chicken caecum. This species includes all bacteria with genomes that show ≥95% average nucleotide identity (ANI) to the type genome for the species, which is available via NCBI Assembly GCA_904420505.1 and to which we have assigned the MAG ID Coassembly_mag_192. The GC content of the type genome is 51.2% and the genome length is 1.83 Mbp.

**Description of *Candidatus* Alloscillospira gen. nov.**

*Candidatus* Alloscillospira (All.os.cil.lo.spi'ra. Gr. masc. adj. *allos* different; N.L. fem. n. *Oscillospira* a bacterial generic name; N.L. fem. n. *Alloscillospira* organism different from but related to the genus *Oscillospira*).

A bacterial genus identified by metagenomic analyses of the chicken caecum. The genus includes all bacteria with genomes that show ≥60% average amino acid identity (AAI) to the genome of the type strain from the type species *Candidatus* Alloscillospira gallinarum. GTDB-Tk/GTDB Release 86 assigns this genus to the order *Oscillospirales* and to the family *Oscillospiraceae*.

**Description of *Candidatus* Alloscillospira gallinarum sp. nov.**

*Candidatus* Alloscillospira gallinarum (gal.li.na'rum. L. gen. pl. n. *gallinarum* of hens).

A bacterial species identified by metagenomic analyses of the chicken caecum. This species includes all bacteria with genomes that show ≥95% average nucleotide identity (ANI) to the type genome for the species, which is available via NCBI Assembly GCA_904420055.1 and to which we have assigned the MAG ID Coassembly_mag_141. The GC content of the type genome is 49.7% and the genome length is 1.63 Mbp.

**Description of *Candidatus* Aristotella gen. nov.**

*Candidatus* Aristotella (A.ris.to.tel'la. N.L. fem. dim. n. *Aristotella* named after Aristotle Greek philosopher who first described the chicken embryo).

A bacterial genus identified by metagenomic analyses of the chicken caecum. The genus includes all bacteria with genomes that show ≥60% average amino acid identity (AAI) to the genome of the type strain from the type species *Candidatus* Aristotella avistercoris. GTDB-Tk/GTDB Release 86 assigns this genus to the order *Oscillospirales* and to the family *Ruminococcaceae*.

**Description of *Candidatus* Aristotella avistercoris sp. nov.**

*Candidatus* Aristotella avistercoris (a.vi.ster'co.ris L. fem. n. *avis* a bird; L. neut. n. *stercus* faeces; N.L. gen. n. *avistercoris* from the faeces of birds).

A bacterial species identified by metagenomic analyses of the chicken caecum. This species includes all bacteria with genomes that show ≥95% average nucleotide identity (ANI) to the type genome for the species, which is available via NCBI Assembly GCA_904397835.1 and to which we have assigned the MAG ID Coassembly_mag_500. The GC content of the type genome is 57.6% and the genome length is 2.22 Mbp.

**Description of *Candidatus* Avimicrobium gen. nov.**

*Candidatus* Avimicrobium (A.vi.mi.cro'bi.um. L. fem. n. *avis* a bird; N.L. neut. n. *microbium* microbe; N.L. neut. n. *Avimicrobium* microbe of birds).

A bacterial genus identified by metagenomic analyses of the chicken caecum. The genus includes all bacteria with genomes that show ≥60% average amino acid identity (AAI) to the genome of the type strain from the type species *Candidatus* Avimicrobium caecorum. GTDB-Tk/GTDB Release 86 assigns this genus to the order *Oscillospirales* and to the family *Ruminococcaceae*.

**Description of *Candidatus* Avimicrobium caecorum sp. nov.**

*Candidatus* Avimicrobium caecorum (cae.co'rum. N.L. neut. n. *caecum* caecum; L. gen. pl. n. *caecorum* of caeca).

A bacterial species identified by metagenomic analyses of the chicken caecum. This species includes all bacteria with genomes that show ≥95% average nucleotide identity (ANI) to the type genome for the species, which is available via NCBI Assembly GCA_904394705.1 and to which we have assigned the MAG ID Coassembly_mag_137. The GC content of the type genome is 57.3% and the genome length is 1.99 Mbp.

**Description of *Candidatus* Avimicrobium faecavium sp. nov.**

*Candidatus* Avimicrobium faecavium (faec.a'vi.um. L. fem. n. *faex* dregs; L. fem. n. *avis* a bird; L. gen. pl. n. *faecavium* from the faeces of birds).

A bacterial species identified by metagenomic analyses of the chicken caecum. This species includes all bacteria with genomes that show ≥95% average nucleotide identity (ANI) to the type genome for the species, which is available via NCBI Assembly GCA_904419105.1 and to which we have assigned the MAG ID Chicken_15_mag_135. The GC content of the type genome is 62% and the genome length is 1.54 Mbp.

**Description of *Candidatus* Avimonas gen. nov.**

*Candidatus* Avimonas (A.vi.mo'nas. L. fem. n. *avis* a bird; Gr. fem. n. *monas* unit monad; N.L. fem. n. *Avimonas* a monad colonising birds).

A bacterial genus identified by metagenomic analyses of the chicken caecum. The genus includes all bacteria with genomes that show ≥60% average amino acid identity (AAI) to the genome of the type strain from the type species *Candidatus* Avimonas caecicola. GTDB-Tk/GTDB Release 86 assigns this genus to the order *Oscillospirales* and to the family DTU089.

**Description of *Candidatus* Avimonas caecicola sp. nov.**

*Candidatus* Avimonas caecicola (cae.ci'co.la. N.L. neut. n. *caecum* caecum; L. suff. *cola*, from L. masc. or fem. n. *incola,* an inhabitant; N.L. fem n. *caecicola* inhabitant of the caecum).

A bacterial species identified by metagenomic analyses of the chicken caecum. This species includes all bacteria with genomes that show ≥95% average nucleotide identity (ANI) to the type genome for the species, which is available via NCBI Assembly GCA_904419875.1 and to which we have assigned the MAG ID Chicken_7_mag_176. The GC content of the type genome is 61% and the genome length is 3.21 Mbp.

**Description of *Candidatus* Avimonas faecium sp. nov.**

*Candidatus* Avimonas faecium (fae'ci.um. L. fem. n. *faex* dregs; L. gen. pl. n. *faecium* of dregs, of faeces).

A bacterial species identified by metagenomic analyses of the chicken caecum. This species includes all bacteria with genomes that show ≥95% average nucleotide identity (ANI) to the type genome for the species, which is available via NCBI Assembly GCA_904419005.1 and to which we have assigned the MAG ID Chicken_12_mag_180. The GC content of the type genome is 61.2% and the genome length is 2.57 Mbp.

**Description of *Candidatus* Avimonas intestinalis sp. nov.**

*Candidatus* Avimonas intestinalis (in.tes.ti.na'lis. N.L. fem. adj. *intestinalis*, pertaining to the intestine).

A bacterial species identified by metagenomic analyses of the chicken caecum. This species includes all bacteria with genomes that show ≥95% average nucleotide identity (ANI) to the type genome for the species, which is available via NCBI Assembly GCA_904380375.1 and to which we have assigned the MAG ID Chicken_18_mag_13. The GC content of the type genome is 60.6% and the genome length is 2.76 Mbp.

**Description of *Candidatus* Avimonas merdigallinarum sp. nov.**

*Candidatus* Avimonas merdigallinarum (mer.di.gal.li.na'rum. L. fem. n. *merda* faeces; L. fem. n. *gallina* a hen; N.L. gen. pl. n. *merdigallinarum* from the faeces of birds).

A bacterial species identified by metagenomic analyses of the chicken caecum. This species includes all bacteria with genomes that show ≥95% average nucleotide identity (ANI) to the type genome for the species, which is available via NCBI Assembly GCA_904377855.1 and to which we have assigned the MAG ID Chicken_16_mag_116. The GC content of the type genome is 59.6% and the genome length is 2.53 Mbp.

**Description of *Candidatus* Avimonas narfiae sp. nov.**

*Candidatus* Avimonas narfiae (nar'fi.ae. N.L. gen. n. *narfiae* named after the National Avian Research Facility where the taxon was first described).

A bacterial species identified by metagenomic analyses of the chicken caecum. This species includes all bacteria with genomes that show ≥95% average nucleotide identity (ANI) to the type genome for the species, which is available via NCBI Assembly GCA_904395335.1 and to which we have assigned the MAG ID Coassembly_mag_196. The GC content of the type genome is 56.1% and the genome length is 2.45 Mbp.

**Description of *Candidatus* Avispirillum gen. nov.**

*Candidatus* Avispirillum (A.vi.spi.ril'lum. L. fem. n. *avis* a bird; N.L. dim. neut. n. *spirillum* a small spiral; N.L. neut. n. *Avispirillum* a small spiral colonising birds).

A bacterial genus identified by metagenomic analyses of the chicken caecum. The genus includes all bacteria with genomes that show ≥60% average amino acid identity (AAI) to the genome of the type strain from the type species *Candidatus* Avispirillum faecium. GTDB-Tk/GTDB Release 86 assigns this genus to the order *Oscillospirales* and to the family CAG-272.

**Description of *Candidatus* Avispirillum faecium sp. nov.**

*Candidatus* Avispirillum faecium (fae'ci.um. L. fem. n. *faex* dregs; L. gen. pl. n. *faecium* of the dregs of faeces).

A bacterial species identified by metagenomic analyses of the chicken caecum. This species includes all bacteria with genomes that show ≥95% average nucleotide identity (ANI) to the type genome for the species, which is available via NCBI Assembly GCA_904396155.1 and to which we have assigned the MAG ID Coassembly_mag_332. The GC content of the type genome is 51.4% and the genome length is 1.95 Mbp.

**Description of *Candidatus* Cryptoclostridium gen. nov.**

*Candidatus* Cryptoclostridium (Cryp.to.clos.tri'di.um. Gr. masc. adj. *kryptos* hidden; N.L. neut. n. *Clostridium* a bacterial generic name; N.L. neut. n. *Cryptoclostridium* a hidden bacterium related to Clostridium).

A bacterial genus identified by metagenomic analyses of the chicken caecum. The genus includes all bacteria with genomes that show ≥60% average amino acid identity (AAI) to the genome of the type strain from the type species *Candidatus* Cryptoclostridium obscurum. This genus has been identified as related to but distinct from the genus *Clostridium* through phylogenetic analysis.

**Description of *Candidatus* Cryptoclostridium obscurum sp. nov.**

*Candidatus* Cryptoclostridium obscurum (ob.scu'rum. L. neut. adj. *obscurum* obscure).

A bacterial species identified by metagenomic analyses of the chicken caecum. This species includes all bacteria with genomes that show ≥95% average nucleotide identity (ANI) to the type genome for the species, which is available via NCBI Assembly GCA_904420535.1 and to which we have assigned the MAG ID Coassembly_mag_106. The GC content of the type genome is 44.6% and the genome length is 2.10 Mbp.

**Description of *Candidatus* Falkowella gen. nov.**

*Candidatus* Falkowella (Fal.kow.el'la. N.L. fem. n. *Falkowella* named in honour of US microbiologist Stanley Falkow).

A bacterial genus identified by metagenomic analyses of the chicken caecum. The genus includes all bacteria with genomes that show ≥60% average amino acid identity (AAI) to the genome of the type strain from the type species *Candidatus* Falkowella caecavicola. GTDB-Tk/GTDB Release 86 assigns this genus to the order *Oscillospirales* and to the family *Ruminococcaceae*.

**Description of *Candidatus* Falkowella caecavicola sp. nov.**

*Candidatus* Falkowella caecavicola (caec.a.vi'co.la. N.L. neut. n. *caecum* caecum; L. fem. n. *avis* a bird; L. suff. *cola*, from L. masc. or fem. n. *incola,* an inhabitant; N.L. fem. n. *caecavicola* inhabits the caecum of birds).

A bacterial species identified by metagenomic analyses of the chicken caecum. This species includes all bacteria with genomes that show ≥95% average nucleotide identity (ANI) to the type genome for the species, which is available via NCBI Assembly GCA_904420125.1 and to which we have assigned the MAG ID Coassembly_mag_370. The GC content of the type genome is 46.6% and the genome length is 2.45 Mbp.

**Description of *Candidatus* Flemingiibacterium gen. nov.**

*Candidatus* Flemingiibacterium (Fle.ming.i.i.bac.te'ri.um. N.L. neut. n. *bacterium* a small rod; N.L. neut. n. *Flemingiibacterium* a rod named after Alexander Fleming, Scottish microbiologist who discovered penicillin).

A bacterial genus identified by metagenomic analyses of the chicken caecum. The genus includes all bacteria with genomes that show ≥60% average amino acid identity (AAI) to the genome of the type strain from the type species *Candidatus* Flemingiibacterium merdigallinarum. GTDB-Tk/GTDB Release 86 assigns this genus to the order *Oscillospirales* and to the family CAG-272

**Description of *Candidatus* Flemingiibacterium merdigallinarum sp. nov.**

*Candidatus* Flemingibacterium merdigallinarum (mer.di.gal.li.na'rum. L. fem. n. *merda* faeces; L. fem. n. *gallina* a hen; N.L. gen. pl. n. *merdigallinarum* from the faeces of birds).

A bacterial species identified by metagenomic analyses of the chicken caecum. This species includes all bacteria with genomes that show ≥95% average nucleotide identity (ANI) to the type genome for the species, which is available via NCBI Assembly GCA_904386005.1 and to which we have assigned the MAG ID Chicken_22_mag_3. The GC content of the type genome is 53.4% and the genome length is 3.49 Mbp.

**Description of *Candidatus* Gallimonas gen. nov.**

*Candidatus* Gallimonas (Gal.li.mo'nas. L. masc. n. *gallus* a chicken; Gr. fem. n. *monas* unit monad; N.L. fem. n. *Gallimonas* a monad colonising chickens).

A bacterial genus identified by metagenomic analyses of the chicken caecum. The genus includes all bacteria with genomes that show ≥60% average amino acid identity (AAI) to the genome of the type strain from the type species *Candidatus* Gallimonas caecicola. GTDB-Tk/GTDB Release 86 assigns this genus to the order 4C28d-15 and to the family CAG-727.

**Description of *Candidatus* Gallimonas caecicola sp. nov.**

*Candidatus* Gallimonas caecicola (cae.ci'co.la. N.L. neut. n. *caecum* caecum; L. suff. *cola*, from L. masc. or fem. n. *incola,* an inhabitant; N.L. masc. n. *caecicola* inhabits the caecum).

A bacterial species identified by metagenomic analyses of the chicken caecum. This species includes all bacteria with genomes that show ≥95% average nucleotide identity (ANI) to the type genome for the species, which is available via NCBI Assembly GCA_904394195.1 and to which we have assigned the MAG ID Chicken_8_mag_76. The GC content of the type genome is 59% and the genome length is 1.37 Mbp.

**Description of *Candidatus* Gallimonas faecavium sp. nov.**

*Candidatus* Gallimonas faecavium (faec.a'vi.um. L. fem. n. *faex* dregs; L. fem. n. *avis* a bird; N.L. gen. pl. n. *faecavium* from the faeces of birds).

A bacterial species identified by metagenomic analyses of the chicken caecum. This species includes all bacteria with genomes that show ≥95% average nucleotide identity (ANI) to the type genome for the species, which is available via NCBI Assembly GCA_904391375.1 and to which we have assigned the MAG ID Chicken_6_mag_57. The GC content of the type genome is 58.6% and the genome length is 1.64 Mbp.

**Description of *Candidatus* Gallimonas faecium sp. nov.**

*Candidatus* Gallimonas faecium (fae'ci.um. L. fem. n. *faex* dregs; L. gen. pl. n. *faecium* of faeces).

A bacterial species identified by metagenomic analyses of the chicken caecum. This species includes all bacteria with genomes that show ≥95% average nucleotide identity (ANI) to the type genome for the species, which is available via NCBI Assembly GCA_904378075.1 and to which we have assigned the MAG ID Chicken_16_mag_149. The GC content of the type genome is 57.2% and the genome length is 1.79 Mbp.

**Description of *Candidatus* Gallimonas intestinalis sp. nov.**

*Candidatus* Gallimonas intestinalis (in.tes.ti.na'lis. N.L. fem. adj. intestinalis, pertaining to the intestine).

A bacterial species identified by metagenomic analyses of the chicken caecum. This species includes all bacteria with genomes that show ≥95% average nucleotide identity (ANI) to the type genome for the species, which is available via NCBI Assembly GCA_904394655.1 and to which we have assigned the MAG ID Chicken_9_mag_69. The GC content of the type genome is 52.9% and the genome length is 1.85 Mbp.

**Description of *Candidatus* Gallimonas merdae sp. nov.**

*Candidatus* Gallimonas merdae (mer'dae. L. gen. n. *merdae* of faeces).

A bacterial species identified by metagenomic analyses of the chicken caecum. This species includes all bacteria with genomes that show ≥95% average nucleotide identity (ANI) to the type genome for the species, which is available via NCBI Assembly GCA_904419525.1 and to which we have assigned the MAG ID Chicken_18_mag_43. The GC content of the type genome is 55.8% and the genome length is 2.08 Mbp.

**Description of *Candidatus* Gallimonas merdigallinarum sp. nov.**

*Candidatus* Gallimonas merdigallinarum (mer.di.gal.li.na'rum. L. fem. n. *merda* faeces referring to source of isolate; L. fem. n. *gallina* hen; N.L. gen. pl. n. *merdigallinarum* from the faeces of birds).

A bacterial species identified by metagenomic analyses of the chicken caecum. This species includes all bacteria with genomes that show ≥95% average nucleotide identity (ANI) to the type genome for the species, which is available via NCBI Assembly GCA_904390485.1 and to which we have assigned the MAG ID Chicken_4_mag_20. The GC content of the type genome is 56.6% and the genome length is 1.72 Mbp.

**Description of *Candidatus* Gallispira gen. nov.**

*Candidatus* Gallispira (Gal.li.spi'ra. L. masc. n. *gallus* a chicken referring to source of isolate; L. fem. n. *spira* a spiral; N.L. fem. n. *Gallispira* a spirillum colonising chickens).

A bacterial genus identified by metagenomic analyses of the chicken caecum. The genus includes all bacteria with genomes that show ≥60% average amino acid identity (AAI) to the genome of the type strain from the type species *Candidatus* Gallispira edinburgensis. GTDB-Tk/GTDB Release 86 assigns this genus to the order *Lachnospirales* and to the family CAG-274

**Description of *Candidatus* Gallispira edinburgensis sp. nov.**

*Candidatus* Gallispira edinburgensis (e.din.burg.en'sis. N.L. fem. adj. *edinburgensis* pertaining to Edinburgh where the taxon was first described referring to source of isolate).

A bacterial species identified by metagenomic analyses of the chicken caecum. This species includes all bacteria with genomes that show ≥95% average nucleotide identity (ANI) to the type genome for the species, which is available via NCBI Assembly GCA_904395805.1 and to which we have assigned the MAG ID Coassembly_mag_263. The GC content of the type genome is 37% and the genome length is 2.50 Mbp.

**Description of *Candidatus* Geddesella gen. nov.**

*Candidatus* Geddesella (Ged.des.el'la. N.L. fem. n. *Geddesella* named after Scottish infectious disease specialist Alasdair Geddes).

A bacterial genus identified by metagenomic analyses of the chicken caecum. The genus includes all bacteria with genomes that show ≥60% average amino acid identity (AAI) to the genome of the type strain from the type species *Candidatus* Geddesella stercoravicola. GTDB-Tk/GTDB Release 86 assigns this genus to the order *Oscillospirales* and to the family UBA644.

**Description of *Candidatus* Geddesella stercoravicola sp. nov.**

*Candidatus* Geddesella stercoravicola (ster.cor.a.vi'co.la. L. neut. n. *stercus* faeces; L. fem. n. *avis* a bird; L. suff. *cola*, from L. masc. or fem. n. *incola,* an inhabitant; N.L. fem. n. *stercoravicola* inhabitant of bird intestinal contents).

A bacterial species identified by metagenomic analyses of the chicken caecum. This species includes all bacteria with genomes that show ≥95% average nucleotide identity (ANI) to the type genome for the species, which is available via NCBI Assembly GCA_904398665.1 and to which we have assigned the MAG ID Coassembly_mag_563. The GC content of the type genome is 39.1% and the genome length is 1.90 Mbp.

**Description of *Candidatus* Harrysmithiimonas gen. nov.**

*Candidatus* Harrysmithiimonas (Har.ry.smith.i.i.mo'nas. Gr. fem. n. *monas* unit monad; N.L. fem. n. *Harrysmithiimonas* a monad named after British microbiologist Harry Smith).

A bacterial genus identified by metagenomic analyses of the chicken caecum. The genus includes all bacteria with genomes that show ≥60% average amin oacid identity (AAI) to the genome of the type strain from the type species *Candidatus* Harrysmithiimonas galli. GTDB-Tk/GTDB Release 86 assigns this genus to the order *Exiguobacterales*.

**Description of *Candidatus* Harrysmithiimonas galli sp. nov.**

*Candidatus* Harrysmithiimonas galli (gal'li. L. gen. n. *galli* of a chicken).

A bacterial species identified by metagenomic analyses of the chicken caecum. This species includes all bacteria with genomes that show ≥95% average nucleotide identity (ANI) to the type genome for the species, which is available via NCBI Assembly GCA_904373265.1 and to which we have assigned the MAG ID Chicken_11_mag_32. The GC content of the type genome is 35.1% and the genome length is 2.70 Mbp.

**Description of *Candidatus* Heritagella gen. nov.**

*Candidatus* Heritagella (He.ri.tag.el'la. N.L. fem. n. *Heritagella* named after UK microbiologist John Heritage).

A bacterial genus identified by metagenomic analyses of the chicken caecum. The genus includes all bacteria with genomes that show ≥60% average amino acid identity (AAI) to the genome of the type strain from the type species *Candidatus* Heritagella caecorum. GTDB-Tk/GTDB Release 86 assigns this genus to the order *Oscillospirales* and to the family DTU089.

**Description of *Candidatus* Heritagella caecorum sp. nov.**

*Candidatus* Heritagella caecorum (cae.co'rum. N.L. neut. n. *caecum* caecum; N.L. gen. n. *caecorum* of the caeca).

A bacterial species identified by metagenomic analyses of the chicken caecum. This species includes all bacteria with genomes that show ≥95% average nucleotide identity (ANI) to the type genome for the species, which is available via NCBI Assembly GCA_904419035.1 and to which we have assigned the MAG ID Chicken_12_mag_185. The GC content of the type genome is 54.6% and the genome length is 2.92 Mbp.

**Description of *Candidatus* Heritagella gallinarum sp. nov.**

*Candidatus* Heritagella gallinarum (gal.li.na'rum. L. gen. pl. n.. *gallinarum* of chickens).

A bacterial species identified by metagenomic analyses of the chicken caecum. This species includes all bacteria with genomes that show ≥95% average nucleotide identity (ANI) to the type genome for the species, which is available via NCBI Assembly GCA_904394685.1 and to which we have assigned the MAG ID Coassembly_mag_139. The GC content of the type genome is 50.6% and the genome length is 2.23 Mbp.

**Description of *Candidatus* Heritagella intestinalis sp. nov.**

*Candidatus* Heritagella intestinalis (in.tes.ti.na'lis. N.L. fem. adj. *intestinalis* pertaining to the intestine).

A bacterial species identified by metagenomic analyses of the chicken caecum. This species includes all bacteria with genomes that show ≥95% average nucleotide identity (ANI) to the type genome for the species, which is available via NCBI Assembly GCA_904418995.1 and to which we have assigned the MAG ID Chicken_13_mag_131. The GC content of the type genome is 55.8% and the genome length is 2.29 Mbp.

**Description of *Candidatus* Heteroclostridium gen. nov.**

*Candidatus* Heteroclostridium (He.te.ro.clos.tri'di.um. Gr. adv. *heteros* different; N.L. neut. n. *Clostridium* a bacterial generic name; N.L. neut. n. *Heteroclostridium* organism different from but related to the genus *Clostridium*).

A bacterial genus identified by metagenomic analyses of the chicken caecum. The genus includes all bacteria with genomes that show ≥60% average amino acid identity (AAI) to the genome of the type strain from the type species *Candidatus* Heteroclostridium caecigallinarum. GTDB-Tk/GTDB Release 86 assigns this genus to the order 4C28d-15 and to the family CAG-314.

**Description of *Candidatus* Heteroclostridium caecigallinarum sp. nov.**

*Candidatus* Heteroclostridium caecigallinarum (cae.ci.gal.li.na'rum. N.L. neut. n. *caecum* caecum; L. fem. n. *gallina* a hen; L. gen. pl. n. *caecigallinarum* from the caeca of chickens).

A bacterial species identified by metagenomic analyses of the chicken caecum. This species includes all bacteria with genomes that show ≥95% average nucleotide identity (ANI) to the type genome for the species, which is available via NCBI Assembly GCA_904419725.1 and to which we have assigned the MAG ID Chicken_24_mag_188. The GC content of the type genome is 51.2% and the genome length is 1.28 Mbp.

**Description of *Candidatus* Heteroruminococcus gen. nov.**

*Candidatus* Heteroruminococcus (He.te.ro.ru.mi.no.coc'cus. Gr. adj. *heteros* different; N.L. masc. n. *Ruminococcus* a bacterial generic name; N.L. masc. n. *Heteroruminococcus* organism different from but related to the genus *Ruminococcus*).

A bacterial genus identified by metagenomic analyses of the chicken caecum. The genus includes all bacteria with genomes that show ≥60% average amino acid identity (AAI) to the genome of the type strain from the type species *Candidatus* Heteroruminococcus faecigallinarum. GTDB-Tk/GTDB Release 86 assigns this genus to the order *Oscillospirales* and to the family *Ruminococcaceae*.

**Description of *Candidatus* Heteroruminococcus faecigallinarum sp. nov.**

*Candidatus* Heteroruminococcus faecigallinarum (fae.ci.gal.li.na'rum. L. fem. n. *faex* dregs; L. fem. n. *gallina* a hens; N.L. gen. pl. n. *faecigallinarum* from the intestinal contents of chickens).

A bacterial species identified by metagenomic analyses of the chicken caecum. This species includes all bacteria with genomes that show ≥95% average nucleotide identity (ANI) to the type genome for the species, which is available via NCBI Assembly GCA_904387055.1 and to which we have assigned the MAG ID Chicken_22_mag_76. The GC content of the type genome is 61.9% and the genome length is 2.30 Mbp.

**Description of *Candidatus* Heteroscillospira gen. nov.**

*Candidatus* Heteroscillospira (He.ter.os.cil.lospi'ra. Gr. adj. *heteros* different; N.L. fem. n. *Oscillospira* a bacterial generic name; N.L. fem. n. *Heteroscillospira* a new organism related to the genus *Oscillospira*).

A bacterial genus identified by metagenomic analyses of the chicken caecum. The genus includes all bacteria with genomes that show ≥60% average amino acid identity (AAI) to the genome of the type strain from the type species *Candidatus* Heteroscillospira lomanii. GTDB-Tk/GTDB Release 86 assigns this genus to the order *Oscillospirales* and to the family *Oscillospiraceae*

**Description of *Candidatus* Heteroscillospira lomanii sp. nov.**

*Candidatus* Heteroscillospira lomanii (lo.man'i.i. N.L. gen. n. *lomanii* named in honour of British microbiologist and bioinformatician Nick Loman).

A bacterial species identified by metagenomic analyses of the chicken caecum. This species includes all bacteria with genomes that show ≥95% average nucleotide identity (ANI) to the type genome for the species, which is available via NCBI Assembly GCA_904419385.1 and to which we have assigned the MAG ID Chicken_16_mag_111. The GC content of the type genome is 60.4% and the genome length is 1.79 Mbp.

**Description of *Candidatus* Howiella gen. nov.**

*Candidatus* Howiella (Ho.wi.el'la. N.L. fem. n. *Howiella* named after Scottish microbiologist James Howie).

A bacterial genus identified by metagenomic analyses of the chicken caecum. The genus includes all bacteria with genomes that show ≥60% average amino acid identity (AAI) to the genome of the type strain from the type species *Candidatus* Howiella intestinavium. GTDB-Tk/GTDB Release 86 assigns this genus to the order *Oscillospirales* and to the family DTU089.

**Description of *Candidatus* Howiella intestinavium sp. nov.**

*Candidatus* Howiella intestinavium (in.tes.tin.a'vi.um. L. neut. n. *intestinum* gut intestine; L. fem. n. *avis* a bird; L. gen. pl. n. *intestinavium* from the intestines of birds).

A bacterial species identified by metagenomic analyses of the chicken caecum. This species includes all bacteria with genomes that show ≥95% average nucleotide identity (ANI) to the type genome for the species, which is available via NCBI Assembly GCA_904420585.1 and to which we have assigned the MAG ID Coassembly_mag_89. The GC content of the type genome is 59.6% and the genome length is 2.43 Mbp.

**Description of *Candidatus* Metalachnospira gen. nov.**

*Candidatus* Metalachnospira (Me.ta.lach.no.spi'ra. Gr. prep. *meta* besides; N.L. fem. n. *Lachnospira* a bacterial generic name; N.L. fem. n. *Metalachnospira* organism related to the genus *Lachnospira*).

A bacterial genus identified by metagenomic analyses of the chicken caecum. The genus includes all bacteria with genomes that show ≥60% average amino acid identity (AAI) to the genome of the type strain from the type species *Candidatus* Metalachnospira gallinarum. GTDB-Tk/GTDB Release 86 assigns this genus to the order *Lachnospirales*.

**Description of *Candidatus* Metalachnospira gallinarum sp. nov.**

*Candidatus* Metalachnospira gallinarum (gal.li.na'rum. L. gen. pl. n. *gallinarum* of hens).

A bacterial species identified by metagenomic analyses of the chicken caecum. This species includes all bacteria with genomes that show ≥95% average nucleotide identity (ANI) to the type genome for the species, which is available via NCBI Assembly GCA_904387325.1 and to which we have assigned the MAG ID Chicken_24_mag_191. The GC content of the type genome is 46.7% and the genome length is 2.08 Mbp.

**Description of *Candidatus* Metaruminococcus gen. nov.**

*Candidatus* Metaruminococcus (Me.ta.ru.mi.no.coc'cus. Gr. prep. *meta* besides; N.L. masc. n. *Ruminococcus* a bacterial generic name; N.L. masc. n. *Metaruminococcus* organism different from but related to the genus *Ruminococcus*).

A bacterial genus identified by metagenomic analyses of the chicken caecum. The genus includes all bacteria with genomes that show ≥60% average amino acid identity (AAI) to the genome of the type strain from the type species *Candidatus* Metaruminococcus caecorum. GTDB-Tk/GTDB Release 86 assigns this genus to the order *Oscillospirales* and to the family *Ruminococcaceae*.

**Description of *Candidatus* Metaruminococcus caecorum sp. nov.**

*Candidatus* Metaruminococcus caecorum (cae.co'rum. N.L. neut. n. *caecum* caecum; L. gen. pl. n. *caecorum* of caeca).

A bacterial species identified by metagenomic analyses of the chicken caecum. This species includes all bacteria with genomes that show ≥95% average nucleotide identity (ANI) to the type genome for the species, which is available via NCBI Assembly GCA_904420045.1 and to which we have assigned the MAG ID Coassembly_mag_161. The GC content of the type genome is 53.6% and the genome length is 2.23 Mbp.

**Description of *Candidatus* Metaruminococcus gallistercoris sp. nov.**

*Candidatus* Metaruminococcus gallistercoris (gal.li.ster'co.ris. L. masc. n *gallus* a chicken; L. neut. n. *stercus* faeces; N.L. gen. n. *gallistercoris* from the intestinal contents of chickens).

A bacterial species identified by metagenomic analyses of the chicken caecum. This species includes all bacteria with genomes that show ≥95% average nucleotide identity (ANI) to the type genome for the species, which is available via NCBI Assembly GCA_904394975.1 and to which we have assigned the MAG ID Coassembly_mag_135. The GC content of the type genome is 48.9% and the genome length is 2.02 Mbp.

**Description of *Candidatus* Neoanaerotignum gen. nov.**

*Candidatus* Neoanaerotignum (Ne.o.an.ae.ro.tig'num. Gr. masc. adj. *neos* new; N.L. neut. n. *Anaerotignum* a bacterial generic name; N.L. neut. n. *Neoanaerotignum* a new organism related to the genus *Anaerotignum*).

A bacterial genus identified by metagenomic analyses of the chicken caecum. The genus includes all bacteria with genomes that show ≥60% average amino acid identity (AAI) to the genome of the type strain from the type species *Candidatus* Neoanaerotignum galli. GTDB-Tk/GTDB Release 86 assigns this genus to the order *Lachnospirales* and to the family *Anaerotignaceae*.

**Description of *Candidatus* Neoanaerotignum galli sp. nov.**

*Candidatus* Neoanaerotignum galli (gal'li. L. gen. n. *galli* of a chicken).

A bacterial species identified by metagenomic analyses of the chicken caecum. This species includes all bacteria with genomes that show ≥95% average nucleotide identity (ANI) to the type genome for the species, which is available via NCBI Assembly GCA_904381335.1 and to which we have assigned the MAG ID Chicken_18_mag_70. The GC content of the type genome is 47.6% and the genome length is 1.85 Mbp.

**Description of *Candidatus* Neoanaerotignum tabaqchaliae sp. nov.**

*Candidatus* Neoanaerotignum tabaqchaliae (ta.baq.cha'li.ae. N.L. gen. n. *tabaqchaliae* named in honour of British/Iraqi microbiologist Soad Tabaqchali).

A bacterial species identified by metagenomic analyses of the chicken caecum. This species includes all bacteria with genomes that show ≥95% average nucleotide identity (ANI) to the type genome for the species, which is available via NCBI Assembly GCA_904420235.1 and to which we have assigned the MAG ID Coassembly_mag_445. The GC content of the type genome is 39% and the genome length is 2.05 Mbp.

**Description of *Candidatus* Neochristensenella gen. nov.**

*Candidatus* Neochristensenella (Ne.o.chris.ten.sen.el'la. Gr. masc. adj. neos *new;* N.L. fem. dim. n. *Christensenella* a bacterial generic name; N.L. fem. n. *Neochristensenella* a new organism related to the genus *Christensenella*).

A bacterial genus identified by metagenomic analyses of the chicken caecum. The genus includes all bacteria with genomes that show ≥60% average amino acid identity (AAI) to the genome of the type strain from the type species *Candidatus* Neochristensenella gallicola. GTDB-Tk/GTDB Release 86 assigns this genus to the order *Christensenellales*.

**Description of *Candidatus* Neochristensenella gallicola sp. nov.**

*Candidatus* Neochristensenella gallicola (gal.li'co.la L. masc. n. *gallus* a chicken; L. suff. *cola*, from L. masc. or fem. n. *incola,* an inhabitant; N.L. masc. n. *gallicola* inhabitant of chicken).

A bacterial species identified by metagenomic analyses of the chicken caecum. This species includes all bacteria with genomes that show ≥95% average nucleotide identity (ANI) to the type genome for the species, which is available via NCBI Assembly GCA_904419735.1 and to which we have assigned the MAG ID Chicken_24_mag_190. The GC content of the type genome is 47% and the genome length is 2.58 Mbp.

**Description of *Candidatus* Neoclostridium gen. nov.**

*Candidatus* Neoclostridium (Ne.o.clos.tri'di.um. Gr. masc. adj. neos *new;* N.L. neut. n. *Clostridium* a bacterial generic name; N.L. neut. n. *Neoclostridium* a new organism related to the genus *Clostridium*).

A bacterial genus identified by metagenomic analyses of the chicken caecum. The genus includes all bacteria with genomes that show ≥60% average amino acid identity (AAI) to the genome of the type strain from the type species *Candidatus* Neoclostridium roslinense. GTDB-Tk/GTDB Release 86 assigns this genus to the order 4C28d-15 and to the family CAG-552.

**Description of *Candidatus* Neoclostridium roslinense sp. nov.**

*Candidatus* Neoclostridium roslinense (ros.lin.en'se. N.L. neut. adj. *roslinense* pertaining to The Roslin Institute where the taxon was first described).

A bacterial species identified by metagenomic analyses of the chicken caecum. This species includes all bacteria with genomes that show ≥95% average nucleotide identity (ANI) to the type genome for the species, which is available via NCBI Assembly GCA_904379005.1 and to which we have assigned the MAG ID Chicken_16_mag_187. The GC content of the type genome is 48.7% and the genome length is 1.52 Mbp.

**Description of *Candidatus* Neoruminococcus gen. nov.**

*Candidatus* Neoruminococcus (Ne.o.ru.mi.no.coc'cus. Gr. masc. adj. neos *new;* N.L. masc. n. *Ruminococcus* a bacterial generic name; N.L. masc. n. *Neoruminococcus* a new organism related to the genus *Ruminococcus*).

A bacterial genus identified by metagenomic analyses of the chicken caecum. The genus includes all bacteria with genomes that show ≥60% average amino acid identity (AAI) to the genome of the type strain from the type species *Candidatus* Neoruminococcus faecicola. GTDB-Tk/GTDB Release 86 assigns this genus to the order *Oscillospirales* and to the family *Ruminococcaceae*.

**Description of *Candidatus* Neoruminococcus faecicola sp. nov.**

*Candidatus* Neoruminococcus faecicola (fae.ci'co.la. L. fem. n. *faex* dregs; L. suff. *cola*, from L. masc. or fem. n. *incola,* an inhabitant; N.L. masc. n. *faecicola* inhabiant of intestinal contents).

A bacterial species identified by metagenomic analyses of the chicken caecum. This species includes all bacteria with genomes that show ≥95% average nucleotide identity (ANI) to the type genome for the species, which is available via NCBI Assembly GCA_904398325.1 and to which we have assigned the MAG ID Coassembly_mag_61. The GC content of the type genome is 61% and the genome length is1.60 Mbp.

**Description of *Candidatus* Parachristensenella gen. nov.**

*Candidatus* Parachristensenella (Pa.ra.chris.ten.sen.el'la. Gr. prep. *para* next to resembling; N.L. fem. dim. n. *Christensenella* a bacterial generic name; N.L. fem. dim. n. *Parachristensenella* organism related to the genus *Christensenella*).

A bacterial genus identified by metagenomic analyses of the chicken caecum. The genus includes all bacteria with genomes that show ≥60% average aminoacid identity (AAI) to the genome of the type strain from the type species *Candidatus* Parachristensenella avicola. GTDB-Tk/GTDB Release 86 assigns this genus to the order *Christensenellales*.

**Description of *Candidatus* Parachristensenella avicola sp. nov.**

*Candidatus* Parachristensenella avicola (a.vi'co.la. L. fem. n. *avis* a bird; L. suff. *cola*, from L. masc. or fem. n. *incola,* an inhabitant; N.L. fem. n. *avicola* an inhabitant of birds).

A bacterial species identified by metagenomic analyses of the chicken caecum. This species includes all bacteria with genomes that show ≥95% average nucleotide identity (ANI) to the type genome for the species, which is available via NCBI Assembly GCA_904420205.1 and to which we have assigned the MAG ID Coassembly_mag_6. The GC content of the type genome is 56.7% and the genome length is 2.96 Mbp.

**Description of *Candidatus* Paralachnospira gen. nov.**

*Candidatus* Paralachnospira (Pa.ra.lach.no.spi'ra. Gr. prep. *para* next to resembling; N.L. fem. n. *Lachnospira* a bacterial generic name; N.L. fem. n. *Paralachnospira* organism related to the genus *Lachnospira*).

A bacterial genus identified by metagenomic analyses of the chicken caecum. The genus includes all bacteria with genomes that show ≥60% average amino acid identity (AAI) to the genome of the type strain from the type species *Candidatus* Paralachnospira avium. GTDB-Tk/GTDB Release 86 assigns this genus to the order *Lachnospirales* and to the family *Lachnospiraceae*.

**Description of *Candidatus* Paralachnospira avium sp. nov.**

*Candidatus* Paralachnospira avium (a'vi.um. L. gen. pl. n. *avium* of birds).

A bacterial species identified by metagenomic analyses of the chicken caecum. This species includes all bacteria with genomes that show ≥95% average nucleotide identity (ANI) to the type genome for the species, which is available via NCBI Assembly GCA_904420245.1 and to which we have assigned the MAG ID Coassembly_mag_381. The GC content of the type genome is 56.1% and the genome length is 2.25 Mbp.

**Description of *Candidatus* Paralachnospira caecorum sp. nov.**

*Candidatus* Paralachnospira caecorum (cae.co'rum. N.L. neut. n. *caecum* caecum; L. gen. pl. n. *caecorum* of caeca).

A bacterial species identified by metagenomic analyses of the chicken caecum. This species includes all bacteria with genomes that show ≥95% average nucleotide identity (ANI) to the type genome for the species, which is available via NCBI Assembly GCA_904379015.1 and to which we have assigned the MAG ID Chicken_16_mag_14. The GC content of the type genome is 52.6% and the genome length is 2.59 Mbp.

**Description of *Candidatus* Paralachnospira sangeri sp. nov.**

*Candidatus* Paralachnospira sangeri (san'ge.ri. N.L. gen. n. *sangeri* named in honour of British scientist Fred Sanger).

A bacterial species identified by metagenomic analyses of the chicken caecum. This species includes all bacteria with genomes that show ≥95% average nucleotide identity (ANI) to the type genome for the species, which is available via NCBI Assembly GCA_904397815.1 and to which we have assigned the MAG ID Coassembly_mag_497. The GC content of the type genome is 51% and the genome length is 3.40 Mbp.

**Description of *Candidatus* Pararuminococcus gen. nov.**

*Candidatus* Pararuminococcus (Pa.ra.ru.mi.no.coc'cus. Gr. prep. *para* next to resembling; N.L. masc. n. *Ruminococcus* a bacterial generic name; N.L. masc. n. *Pararuminococcus* organism related to the genus *Ruminococcus*).

A bacterial genus identified by metagenomic analyses of the chicken caecum. The genus includes all bacteria with genomes that show ≥60% average amino acid identity (AAI) to the genome of the type strain from the type species *Candidatus* Pararuminococcus gallinarum. GTDB-Tk/GTDB Release 86 assigns this genus to the order *Oscillospirales* and to the family *Ruminococcaceae*.

**Description of *Candidatus* Pararuminococcus gallinarum sp. nov.**

*Candidatus* Pararuminococcus gallinarum (gal.li.na'rum. L. gen. pl. n. *gallinarum* of hens).

A bacterial species identified by metagenomic analyses of the chicken caecum. This species includes all bacteria with genomes that show ≥95% average nucleotide identity (ANI) to the type genome for the species, which is available via NCBI Assembly GCA_904419315.1 and to which we have assigned the MAG ID Chicken_15_mag_192. The GC content of the type genome is 50.7% and the genome length is 2.20 Mbp.

**Description of *Candidatus* Protoclostridium gen. nov.**

*Candidatus* Protoclostridium (Pro.to.clos.tri'di.um. Gr. masc. adj. *protos* first; N.L. neut. n. *Clostridium* a bacterial generic name; N.L. neut. n. *Protoclostridium* a new organism related to the genus *Clostridium*).

A bacterial genus identified by metagenomic analyses of the chicken caecum. The genus includes all bacteria with genomes that show ≥60% average amino acid identity (AAI) to the genome of the type strain from the type species *Candidatus* Protoclostridium gallicola. GTDB-Tk/GTDB Release 86 assigns this genus to the order 4C28d-15 and to the family CAG-552.

**Description of *Candidatus* Protoclostridium gallicola sp. nov.**

*Candidatus* Protoclostridium gallicola (gal.li'co.la. L. masc. n. *gallus* a chicken; L. suff. *cola*, from L. masc. or fem. n. *incola*, an inhabitant; N.L. n. *gallicola* an inhabitant of chickens).

A bacterial species identified by metagenomic analyses of the chicken caecum. This species includes all bacteria with genomes that show ≥95% average nucleotide identity (ANI) to the type genome for the species, which is available via NCBI Assembly GCA_904387375.1 and to which we have assigned the MAG ID Chicken_24_mag_120. The GC content of the type genome is 56% and the genome length is 1.80 Mbp.

**Description of *Candidatus* Protoclostridium intestinavium sp. nov.**

*Candidatus* Protoclostridium intestinavium (in.tes.tin.a'vi.um. L. neut. n. *intestinum* gut intestine; L. fem n. *avis* a bird; N.L. gen. pl. n. *intestinavium* from the intestines of birds).

A bacterial species identified by metagenomic analyses of the chicken caecum. This species includes all bacteria with genomes that show ≥95% average nucleotide identity (ANI) to the type genome for the species, which is available via NCBI Assembly GCA_904379215.1 and to which we have assigned the MAG ID Chicken_16_mag_213. The GC content of the type genome is 52.7% and the genome length is 2.06 Mbp.

**Description of *Candidatus* Pseudobutyricicoccus gen. nov.**

*Candidatus* Pseudobutyricicoccus (Pseu.do.bu.ty.ri.ci.coc'cus. Gr. masc. adj. *pseudês* false; N.L. masc. n. *Butyricicoccus* a bacterial generic name; N.L. masc. n. *Pseudobutyricicoccus* a new organism related to the genus *Butyricicoccus*).

A bacterial genus identified by metagenomic analyses of the chicken caecum. The genus includes all bacteria with genomes that show ≥60% average amino acid identity (AAI) to the genome of the type strain from the type species *Candidatus* Pseudobutyricicoccus lothianensis. GTDB-Tk/GTDB Release 86 assigns this genus to the order *Oscillospirales* and to the family *Butyricicoccaceae*.

**Description of *Candidatus* Pseudobutyricicoccus lothianensis sp. nov.**

*Candidatus* Pseudobutyricicoccus lothianensis (lo.thi.an.en'sis. N.L. masc. n. *lothianensis* named after the Lothians where the taxon was first described).

A bacterial species identified by metagenomic analyses of the chicken caecum. This species includes all bacteria with genomes that show ≥95% average nucleotide identity (ANI) to the type genome for the species, which is available via NCBI Assembly GCA_904419295.1 and to which we have assigned the MAG ID Chicken_14_mag_102. The GC content of the type genome is 57.9% and the genome length is 2.62 Mbp.

**Description of *Candidatus* Pseudolachnospira gen. nov.**

*Candidatus* Pseudolachnospira (Pseu.do.lach.no.spi'ra. Gr. masc. adj. *pseudês* false; N.L. fem. n. *Lachnospira* a bacterial generic name; N.L. fem. n. *Pseudolachnospira* a new organism related to the genus *Lachnospira*).

A bacterial genus identified by metagenomic analyses of the chicken caecum. The genus includes all bacteria with genomes that show ≥60% average amino acid identity (AAI) to the genome of the type strain from the type species *Candidatus* Pseudolachnospira avium. GTDB-Tk/GTDB Release 86 assigns this genus to the order *Lachnospirales* and to the family *Lachnospiraceae*.

**Description of *Candidatus* Pseudolachnospira avium sp. nov.**

*Candidatus* Pseudolachnospira avium (a'vi.um. L. gen. pl. n. *avium* of birds).

A bacterial species identified by metagenomic analyses of the chicken caecum. This species includes all bacteria with genomes that show ≥95% average nucleotide identity (ANI) to the type genome for the species, which is available via NCBI Assembly GCA_904420025.1 and to which we have assigned the MAG ID Coassembly_mag_265. The GC content of the type genome is 58% and the genome length is 3.07 Mbp.

**Description of *Candidatus* Pseudoruminococcus gen. nov.**

*Candidatus* Pseudoruminococcus (Pseu.do.ru.mi.no.coc'cus. Gr. adj. masc. *pseudês* false; N.L. masc. n. *Ruminococcus* a bacterial generic name; N.L. masc. n. *Pseudoruminococcus* a new organism related to the genus *Ruminococcus*).

A bacterial genus identified by metagenomic analyses of the chicken caecum. The genus includes all bacteria with genomes that show ≥60% average amino acid identity (AAI) to the genome of the type strain from the type species *Candidatus* Pseudoruminococcus merdavium. GTDB-Tk/GTDB Release 86 assigns this genus to the order *Oscillospirales* and to the family *Ruminococcaceae*.

**Description of *Candidatus* Pseudoruminococcus merdavium sp. nov.**

*Candidatus* Pseudoruminococcus merdavium (merd.a'vi.um L. fem. n. *merda* faeces; N.L. fem. n. *avis* a bird; L. gen. pl. n. *merdavium* from the faeces of birds).

A bacterial species identified by metagenomic analyses of the chicken caecum. This species includes all bacteria with genomes that show ≥95% average nucleotide identity (ANI) to the type genome for the species, which is available via NCBI Assembly GCA_904419745.1 and to which we have assigned the MAG ID Chicken_3_mag_121. The GC content of the type genome is 46.3% and the genome length is 2.24 Mbp.

**Description of *Candidatus* Pseudoscillospira gen. nov.**

*Candidatus* Pseudoscillospira (Pseud.os.cil.lo.spi'ra. Gr. masc. adj. *pseudês* false; N.L. fem. n. *Oscillospira* a bacterial generic name; N.L. fem. n. *Pseudoscillospira* a new organism related to the genus *Oscillospira*).

A bacterial genus identified by metagenomic analyses of the chicken caecum. The genus includes all bacteria with genomes that show ≥60% average amino acid identity (AAI) to the genome of the type strain from the type species *Candidatus* Pseudoscillospira faecavium. GTDB-Tk/GTDB Release 86 assigns this genus to the order *Oscillospirales* and to the family *Oscillospiraceae*.

**Description of *Candidatus* Pseudoscillospira faecavium sp. nov.**

*Candidatus* Pseudoscillospira faecavium (faec.a'vi.um. L. fem. n. *faex* dregs; L. fem. n. *avid* a bird; N.L. gen. pl. n. *faecavium* from the faeces of birds).

A bacterial species identified by metagenomic analyses of the chicken caecum. This species includes all bacteria with genomes that show ≥95% average nucleotide identity (ANI) to the type genome for the species, which is available via NCBI Assembly GCA_904420565.1 and to which we have assigned the MAG ID Coassembly_mag_557. The GC content of the type genome is 53.2% and the genome length is 1.83 Mbp.

**Description of *Candidatus* Pseudoscillospira falkowii sp. nov.**

*Candidatus* Pseudoscilispira falkowii (fal.kow'i.i. N.L. gen. n. *falkowii* named in honour of US microbiologist Stanley Falkow).

A bacterial species identified by metagenomic analyses of the chicken caecum. This species includes all bacteria with genomes that show ≥95% average nucleotide identity (ANI) to the type genome for the species, which is available via NCBI Assembly GCA_904374485.1 and to which we have assigned the MAG ID Chicken_12_mag_143. The GC content of the type genome is 61.6% and the genome length is 1.89 Mbp.

**Description of *Candidatus* Roslinia gen. nov.**

*Candidatus* Roslinia (Ros.li'ni.a. N.L. fem. n. *Roslinia* named after the Roslin Institute where the genus was discovered).

A bacterial genus identified by metagenomic analyses of the chicken caecum. The genus includes all bacteria with genomes that show ≥60% average amino acid identity (AAI) to the genome of the type strain from the type species *Candidatus* Roslinia caecavium. GTDB-Tk/GTDB Release 86 assigns this genus to the order CAG-41.

**Description of *Candidatus* Roslinia caecavium sp. nov.**

*Candidatus* Roslinia caecavium (caec.a'vi.um. N.L. neut. n. *caecum* caecum; L. fem. n. *avis* a bird; N.L. gen. pl. n. *caecavium* from the caeca of birds).

A bacterial species identified by metagenomic analyses of the chicken caecum. This species includes all bacteria with genomes that show ≥95% average nucleotide identity (ANI) to the type genome for the species, which is available via NCBI Assembly GCA_904419955.1 and to which we have assigned the MAG ID Chicken_8_mag_97. The GC content of the type genome is 47.8% and the genome length is 2.81 Mbp.

**Description of *Candidatus* Schneewindia gen. nov.**

*Candidatus* Schneewindia (Schnee.win'di.a N.L. fem. n. *Schneewindia* named after the German-American microbiologist Olaf Schneewind).

A bacterial genus identified by metagenomic analyses of the chicken caecum. The genus includes all bacteria with genomes that show ≥60% average amino acid identity (AAI) to the genome of the type strain from the type species *Candidatus* Schneewindia gallinarum. GTDB-Tk/GTDB Release 86 assigns this genus to the order *Oscillospirales* and to the family *Ruminococcaceae*.

**Description of *Candidatus* Schneewindia gallinarum sp. nov.**

*Candidatus* Schneewindia gallinarum (gal.li.na'rum. L. gen. pl. n. *gallinarum* of hens).

A bacterial species identified by metagenomic analyses of the chicken caecum. This species includes all bacteria with genomes that show ≥95% average nucleotide identity (ANI) to the type genome for the species, which is available via NCBI Assembly GCA_904420175.1 and to which we have assigned the MAG ID Coassembly_mag_388. The GC content of the type genome is 56.6% and the genome length is 1.77 Mbp.

**Description of *Candidatus* Tabaqchalia gen. nov.**

*Candidatus* Tabaqchalia (Ta.baq.cha'li.a. N.L. fem. n. *Tabaqchalia* named after British-Iraqi microbiologist Soad Tabaqchali).

A bacterial genus identified by metagenomic analyses of the chicken caecum. The genus includes all bacteria with genomes that show ≥60% average amino acid identity (AAI) to the genome of the type strain from the type species *Candidatus* Tabaqchalia intestinavium. GTDB-Tk/GTDB Release 86 assigns this genus to the order Oscillospirales.

**Description of *Candidatus* Tabaqchalia intestinavium sp. nov.**

*Candidatus* Tabaqchalia intestinavium (in.tes.tin.a'vi.um. L. neut. n. *intestinum* gut intestine; L. fem. n. *avis* a bird; N.L. gen. pl. n. *intestinavium* from the intestines of birds).

A bacterial species identified by metagenomic analyses of the chicken caecum. This species includes all bacteria with genomes that show ≥95% average nucleotide identity (ANI) to the type genome for the species, which is available via NCBI Assembly GCA_904420005.1 and to which we have assigned the MAG ID Coassembly_mag_113. The GC content of the type genome is 60.8% and the genome length is 2.13 Mbp.

**Description of *Candidatus* Timburyella gen. nov.**

*Candidatus* Timburyella (Tim.bu.ry.el'la. N.L. fem. n. *Timburyella* named after the Scottish microbiologist Morag Timbury).

A bacterial genus identified by metagenomic analyses of the chicken caecum. The genus includes all bacteria with genomes that show ≥60% average amino acid identity (AAI) to the genome of the type strain from the type species *Candidatus* Timburyella stercoris. GTDB-Tk/GTDB Release 86 assigns this genus to the order *Oscillospirales* and to the family DTU089.

**Description of *Candidatus* Timburyella stercoris sp. nov.**

*Candidatus* Timburyella stercoris (ster'co.ris. L. gen. n. *stercoris* of faeces).

A bacterial species identified by metagenomic analyses of the chicken caecum. This species includes all bacteria with genomes that show ≥95% average nucleotide identity (ANI) to the type genome for the species, which is available via NCBI Assembly GCA_904384255.1 and to which we have assigned the MAG ID Chicken_2_mag_40. The GC content of the type genome is 41.7% and the genome length is 2.02 Mbp.

**Description of *Candidatus* Woodwardiibium gen. nov.**

*Candidatus* Woodwardiibium (Wood.ward.i.i'bi.um. Gr. masc. n. *bios* life; N.L. neut. n. *Woodwardiibium* a life form named after Martin Woodward, recognized for his contribution to microbiology).

A bacterial genus identified by metagenomic analyses of the chicken caecum. The genus includes all bacteria with genomes that show ≥60% average amino acid identity (AAI) to the genome of the type strain from the type species *Candidatus* Woodwardiibium gallinarum. GTDB-Tk/GTDB Release 86 assigns this genus to the order *Oscillospirales* and to the family CAG-382i

**Description of *Candidatus* Woodwardiibium gallinarum sp. nov.**

*Candidatus* Woodwardibium gallinarum (gal.li.na'rum. L. gen. pl. n. *gallinarum* of hens)

A bacterial species identified by metagenomic analyses of the chicken caecum. This species includes all bacteria with genomes that show ≥95% average nucleotide identity (ANI) to the type genome for the species, which is available via NCBI Assembly GCA_904419545.1 and to which we have assigned the MAG ID Chicken_20_mag_147. The GC content of the type genome is 55.4% and the genome length is 2.32 Mbp.
